# Supplementary material for: Anti-Freezing Nanocomposite Organohydrogels with High Strength and Toughness
Source: Polymers (Basel). 2022 Sep 6;14(18):3721. doi: 10.3390/polym14183721 (PMC9500911; doi:10.3390/polym14183721)
Supplement: Supplementary file 1 [file polymers-14-03721-s001.zip › Supplementary Files-Proofread.pdf]

polymers

Supplementary Materials: Anti-Freezing Nanocomposite Organohydrogels with High Strength and Toughness

Huijuan Zheng, Qiqi Huang, Meijun Lu, Jiaxin Fu, Zhen Liang, Tong Zhang, Di Wang * and Chengpeng Li

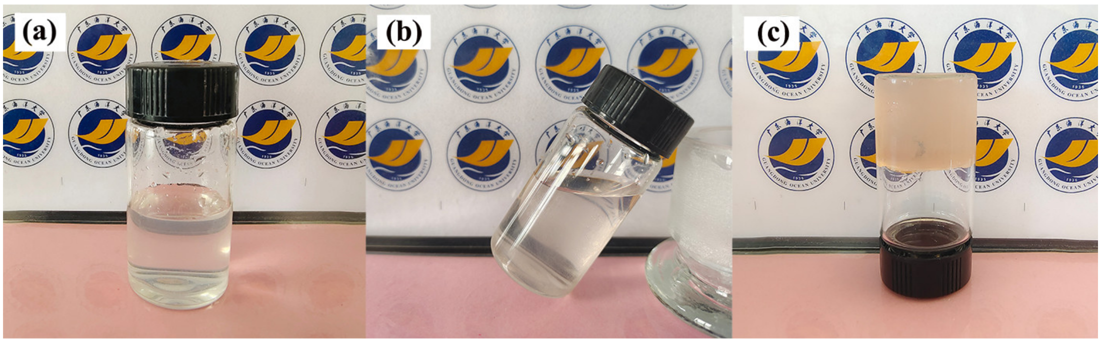

Figure S1. Optical images of the preparation process of the pre-hydrogels with the molar ratio of NIPAM/HEA of 5:5. (a) dispersion of Laponite and deionized water; (b) prepolymer solution; (c) prepolymer solution which gelled.
